# A Critical Review of Spatial Abilities in Down and Williams Syndromes: Not All Space Is Created Equal

**DOI:** 10.3389/fpsyt.2021.669320

**Published:** 2021-05-28

**Authors:** Pamela Banta Lavenex, Pierre Lavenex

**Affiliations:** ^1^Faculty of Psychology, UniDistance Suisse, Brig, Switzerland; ^2^Institute of Psychology, University of Lausanne, Lausanne, Switzerland

**Keywords:** egocentric, homing behavior, allocentric, cognitive map, spatial memory, navigation, neurodevelopmental disorders

## Abstract

Down syndrome (DS, Trisomy 21) and Williams syndrome (WS) are two neurodevelopmental disorders of genetic origin that are accompanied by mild to moderate intellectual disability but exhibit distinct cognitive profiles. In this review we discuss our recent work characterizing the real-world spatial learning and memory abilities of adult individuals with DS and WS. We used several different paradigms in which participants locomote freely and have access to coherent input from all sensory modalities to investigate their fundamental egocentric (body-centered or viewpoint-dependent) and allocentric (world-centered or viewpoint-independent) spatial abilities. We found unequivocal evidence that most individuals with DS exhibit low-resolution egocentric and allocentric spatial learning and memory abilities similar to typically developing (TD) children in the same mental age range. In contrast, most individuals with DS exhibit impaired high-resolution allocentric spatial learning and facilitated response learning as compared to TD children. In comparison, whereas most individuals with WS also exhibit facilitated response learning, their low-resolution allocentric spatial learning and memory abilities are severely impaired as compared to both TD children and individuals with DS. Together with work from other laboratories using real-world or virtual reality paradigms, these findings indicate that in order to navigate in their environment most individuals with DS may use either egocentric route learning that does not integrate individual landmarks, or a low-resolution allocentric spatial representation that encodes the relationships between different locations (i.e., cognitive mapping). In contrast, since most individuals with WS are unable to build or use a low-resolution allocentric or configural representation of the environment they may use visually and verbally encoded landmarks as beacons to learn routes. Finally, we discuss the main neural structures implicated in these different spatial processes and explain how the relative preservation or impairment of specific brain functions may engender the unique cognitive profiles observed in individuals with these neurodevelopmental disorders.

## Introduction

Down syndrome (DS, Trisomy 21) and Williams syndrome (WS) are two neurodevelopmental disorders of genetic origin accompanied by mild to moderate intellectual disability. Despite the fact that individuals with DS and WS have relatively similar IQs [DS: mean 50, range 30–70 (1); WS: mean 55, range 40–70 (2)], these two syndromes are characterized by distinct cognitive profiles. Indeed, these syndromes are often described as having opposing profiles with capacities that may be considered strengths in one of the syndromes being considered a weakness in the other (3–5). This has been reported especially for spatial cognition (3, 6, 7). However, spatial cognition is a vast domain that includes many different types of capacities subserved by distinct and dissociable functional brain circuits.

Most scientists now agree that there are two fundamental, neurobiologically-distinct spatial coding systems in the brain (8–12). The egocentric coding system creates spatial representations in which locations are encoded in relation to the observer in a frame of reference centered on the observer's eyes, head, or entire body and also referred to as viewpoint-dependent. Note that this term applies even when visual processes are not necessarily implicated, such as in the dark or in blind individuals, and thus refers more specifically to the idea that the representation is centered on the individual (13, 14). Different brain regions contribute to the processing of egocentric spatial representations but the parietal and parahippocampal cortices are key structures (15, 16). The allocentric coding system, in contrast, creates spatial representations in which locations are encoded in relation to other objects or locations in the environment in a frame of reference centered on the environment and referred to as viewpoint-independent or world-centered (13, 14). Allocentric spatial processing has been shown to depend on the hippocampal formation in rats, monkeys, and humans (17–19). As discussed elsewhere (20), the terms allocentric spatial representations and cognitive maps are considered by most experimental psychologists and neuroscientists to be synonymous (21), and this type of spatial representation supports our ability to know where things are in relation to other things and in turn enables us to navigate flexibly via novel paths or trajectories.

Researchers have relied on two different types of learning paradigms to bring into evidence these different memory systems: (1) Place learning, which requires the ability to use an allocentric representation to learn and remember *where* to find a location or place in the environment. Place learning implicates the hippocampal formation and other related brain structures (22, 23); and (2) Response learning [a.k.a. habit learning (23) or orientation hypothesis as part of the taxon systems (22)], which requires the ability to use an egocentrically defined fixed motor response in order to learn and remember *how* to find a location or place in the environment. In addition to implicating parietal brain regions, the execution of fixed motor responses involves the dorsal striatum (8) and cerebellum (24). Interestingly, work in both rodents and humans has shown that these two memory systems, the hippocampus-dependent place learning system and the striatum-dependent response learning system, can sometimes function in a competitive rather than a cooperative manner (9, 25, 26) and inhibiting the activity of one of these two systems can result in the facilitation of the behavioral response generated by the other system.

Although prior to our recent studies no previously published study had unequivocally shown the preservation or impairment of either egocentric or allocentric spatial memory capacities in DS or WS, the importance of characterizing basic spatial memory capacities in individuals with intellectual disability are manifold. First, since allocentric spatial representations enable flexible behavior, the ability to create and use such representations is particularly important for developing independence and autonomy. Second, allocentric spatial memory is a fundamental component of episodic memory and thus may serve as a proxy for assessing episodic memory function, especially in individuals with impaired language function. Third, because allocentric spatial memory is one of the hallmark cognitive processes studied in mouse models of DS and WS, clarifying the state of this cognitive process in humans with WS and DS is critical in order to validate rodent models. Fourth, comparing the spatial capacities of individuals with DS and WS can identify cognitive deficits that are syndrome-specific and not just due to general intellectual disability, thus highlighting genes and brain structures that are implicated in these processes. In sum, understanding the relative preservation or impairment of egocentric and allocentric spatial capacities in individuals with these two different genetic syndromes is not only important for helping these individuals and their caregivers to adopt appropriate learning strategies to augment autonomy, but may also provide critical information concerning brain structure-function relationships in both typical and atypical development.

## Recent Findings From Our Laboratory

### High-Resolution Allocentric Spatial Capacities in DS

**Figure 1:**
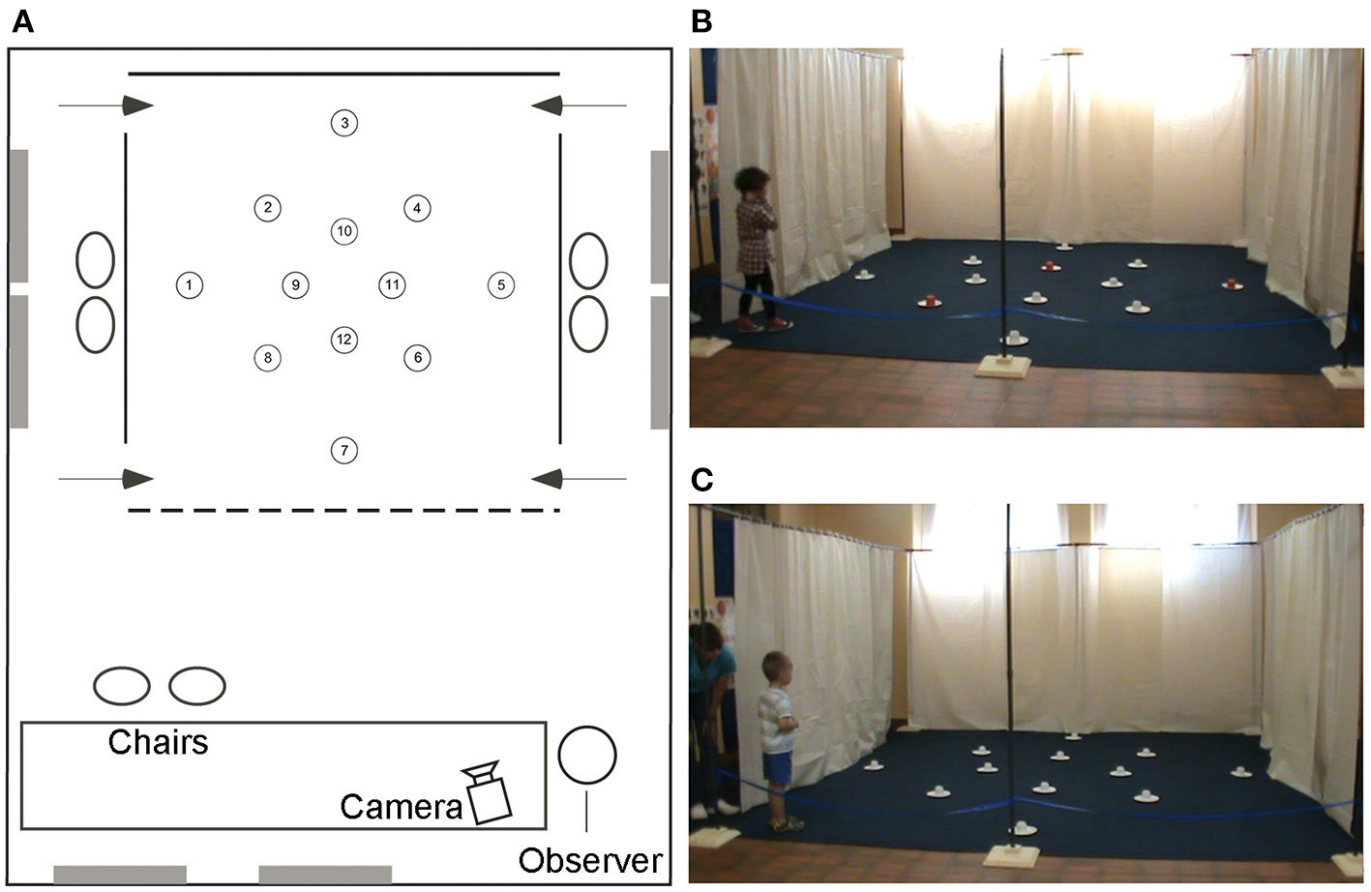
The high-resolution open-field arena. Schematic representation **(A)** and photos **(B,C)** of the open-field arena with 12 potentially rewarded locations. The rewards are hidden under the cups at locations 5, 8, and 10 in both the local cue condition [**(B)** with the red cup] and the allocentric spatial condition [**(C)** all white cups]. The 4 × 4 m arena is placed in a larger testing room containing distal objects that are visible from inside the arena and from the intertrial waiting areas on the left and right sides of the arena. Figure modified from Banta Lavenex et al. (31).

When we began our investigations to characterize the allocentric spatial capacities of individuals with DS only one other study had investigated the spatial capacities of this population in a real-world environment. In his unpublished doctoral studies, Mangan studied the response learning, cue learning, and place learning abilities of children with DS and TD children from 16 to 28 months of age (27). Although a difference in the performance of children with DS and TD children was found in the allocentric place learning task, the fact that allocentric spatial abilities only emerge in TD children around 24 months of age (28–30) left open the possibility that the emergence of allocentric spatial abilities are only delayed in young children with DS and that they may continue to develop normally although perhaps with a slower time course than in TD children. We thus began by investigating the allocentric spatial capacities of developmentally mature individuals with DS using a real-world paradigm (as opposed to paradigms conducted in virtual reality) that we refer to as the open-field paradigm (Figure 1).

Previous studies in our laboratory had shown that from 3.5 years of age TD children can use an allocentric representation to learn and remember with above chance probability the location of three rewarded locations amongst 18 potentially rewarded locations in the open-field paradigm (29). Since the mental age of individuals with DS typically ranges from 5 to 9 years of age we reasoned that a similar paradigm was a logical place to start. We tested the abilities of 20 individuals with DS (mean CA 18.81 years, mean MA 5.3 years) and 16 TD children (mean CA 4.91 years, mean MA 4.97 years) to learn and remember the locations of three rewards amongst 12 potentially rewarded locations (Figure 1).

**Figure 2:**
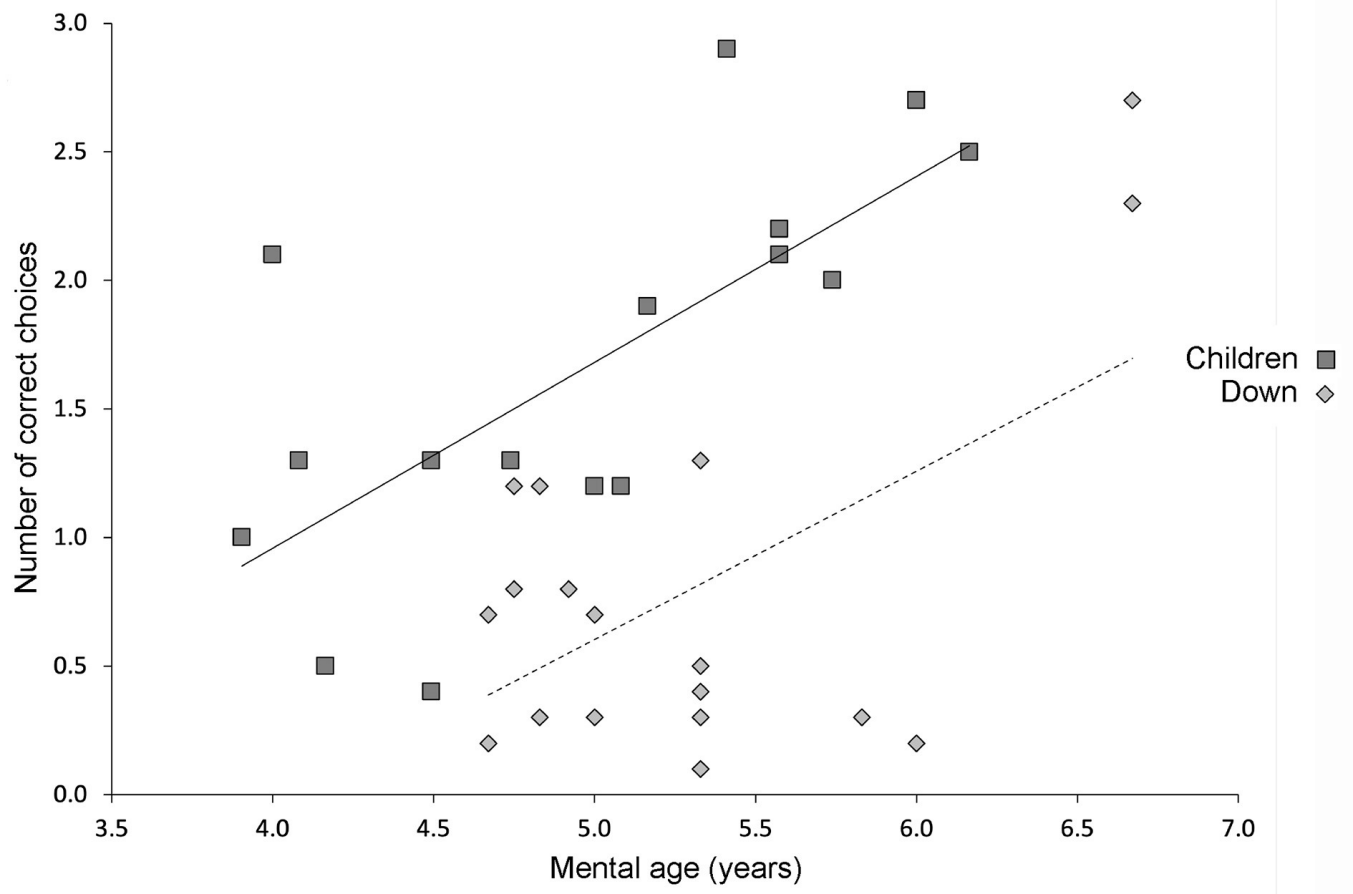
Performance on the high-resolution open-field arena with 12 potentially rewarded locations. Relationship between mental age and performance on the high-resolution open-field arena (illustrated in Figure 1) as assessed by the number of correct choices before erring in TD children and participants with DS. Solid line indicates the average performance of TD children. Dashed line indicates the average performance of individuals with DS. Figure modified from Banta Lavenex et al. (31).

We found that individuals with DS performed similarly to TD children when local cues marked the reward locations (Figure 1A), confirming that they understood the task and were motivated to participate. In contrast, the majority of individuals with DS were impaired in the AS condition, in the absence of local cues (Figure 1B), as compared to TD children (Figure 2). When considering the number of correct choices that participants made before committing their first error, a proxy for memory capacity, all but two individuals had scores below the average of the group of TD children in the same mental age range. Nevertheless, individual analyses showed that there was significant individual variability and that whereas 50% (10/20) of the individuals with DS were incapable of reliably identifying any of the three rewarded locations during AS trials, the other 50% of the individuals discriminated some of the rewarded locations at above chance levels, and two individuals with DS were even capable of reliably finding all three rewards before making an error in the AS condition. Interestingly, these two individuals had the highest mental ages (>6.5 years of age).

The 10 individuals with DS who performed above chance level exhibited a preference for searching location 5 for their first choice after entering the arena. This finding was revealing because, as is discussed below, in recent years our work and the work of others has provided behavioral and neurological evidence that different spatial competencies, subserved by distinct hippocampal circuits, are necessary for low-resolution “topological” and high-resolution “metric” allocentric spatial coding (32–35). Considering this task with 12 potentially rewarded locations, whereas locations 8 and 10 require the use of a high-resolution metric representation in order to be discriminated from surrounding decoy locations, location 5 may be discriminated using a topological representation. Thus, although overall performance of individuals with DS was impaired as compared to TD children, the fact that 50% of the participants with DS preferentially chose location 5 for their first choice (which contributed to their overall above chance performance) suggested that their low-resolution allocentric spatial capacities were relatively preserved. We set out to test this hypothesis specifically with our next series of experiments.

### Low-Resolution Allocentric Spatial Capacities in DS and WS

**Figure 3:**
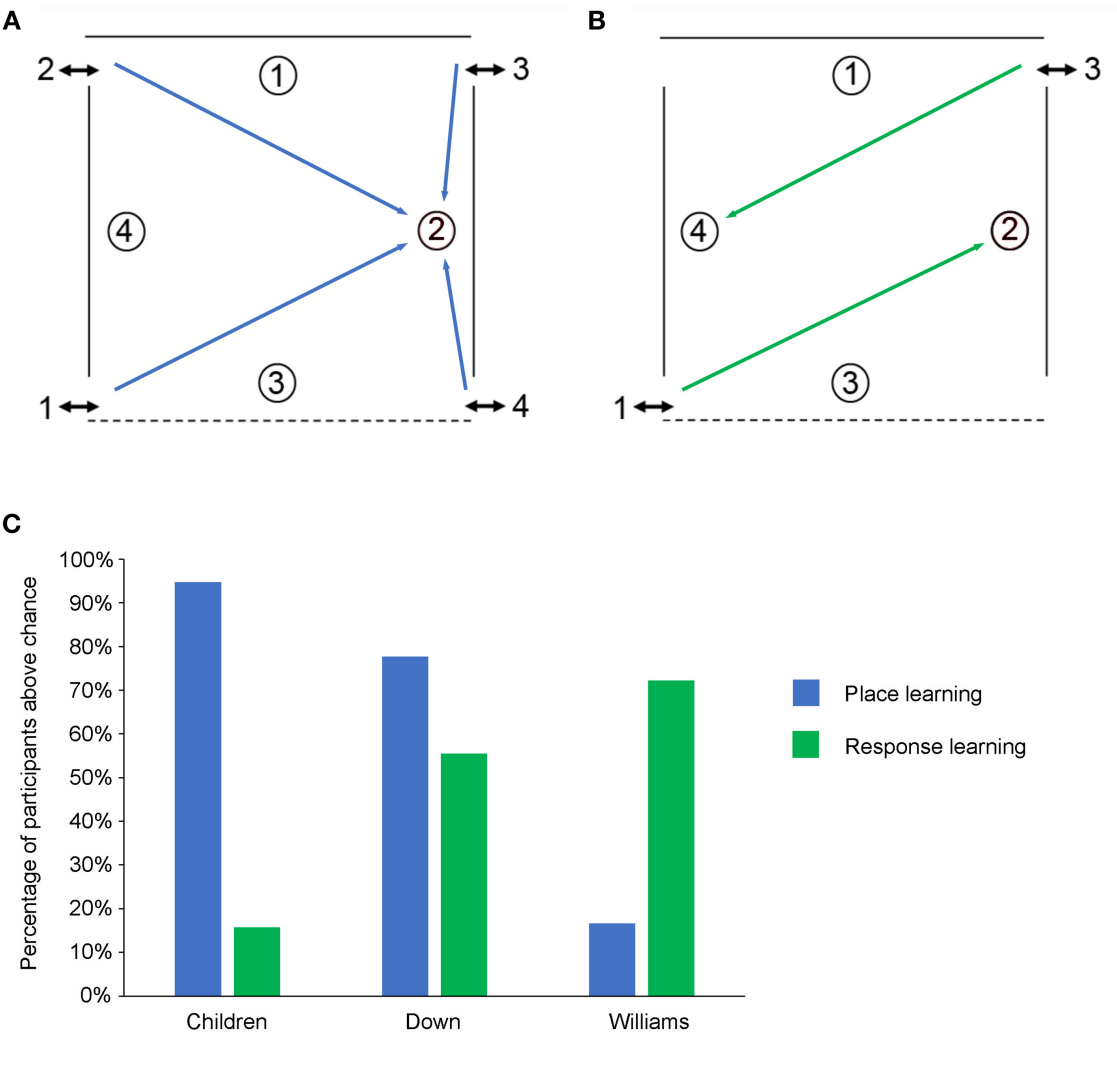
The low-resolution allocentric spatial and response learning tasks. **(A)** Schematic representation of the open-field arena used for testing place learning. The reward was always placed at the same location within the arena. **(B)** Schematic representation of the open-field arena used for testing response learning. The reward could be found by performing the same motor response upon entering the arena. **(C)** Percentage of individuals from the three experimental groups (TD children, individuals with DS, individuals with WS) passing the place learning task and the response learning task.

We tested the abilities of 27 individuals with DS (mean CA 23.4 years, mean MA 5.6 years), 18 individuals with WS (mean CA 21.5 years, mean MA 5.9 years), and 19 TD children (mean CA 5.5 years, mean MA 6.6 years) to learn and remember the location of one reward amongst four potentially rewarded locations [Figure 3A; (36, 37)]. As described above, when the arena contains 12 potentially rewarded locations, high-resolution allocentric metric coding is necessary to learn and remember some of the reward locations. In contrast, when the arena contains only four potentially rewarded locations, and when each location is two or more meters from the others, low-resolution allocentric topological coding is likely sufficient to spatially discriminate these locations.

In the local cue (LC) condition where a red cup signaled the reward location all three groups performed similarly and the number of individuals from each group who performed above chance did not differ. In contrast, in the allocentric spatial (AS) condition (a.k.a. place learning), whereas 95% (18/19) of the TD children and 74% (23/31) of the individuals with DS performed above chance only 16% (3/18) of the WS individuals did so (Figure 3C). The number of TD children and individuals with DS who performed above chance did not differ but fewer individuals with WS passed as compared to the other two groups.

Our previous studies documenting the emergence and development of allocentric spatial memory capacities in TD children using the same paradigm showed that these abilities emerge around 24 months of age in TD children and that by 36 months of age 95% of TD children can solve this task (29, 30). These results thus suggest that individuals with WS are significantly impaired in even the most basic allocentric spatial tasks such as those solved by 2–3-year-old TD children. In contrast, the majority of individuals with DS exhibited basic allocentric spatial abilities suggesting that this capacity is relatively preserved in this population, and confirming the hypothesis formulated after our first experiment.

### Low-Resolution Egocentric Response Learning in DS and WS

As discussed above, the functional brain circuits that underlie egocentric and allocentric spatial representations are distinct and dissociable (8–12). Moreover, it has been shown that inhibiting the activity of one of these two systems can result in the facilitation of the behavioral response supported by the other system (9, 25, 26). Since individuals with WS and individuals with DS exhibited deficits on hippocampal-dependent spatial learning tasks, we were interested in investigating egocentric response learning in these same individuals using a modified procedure in the open-field arena. As for low-resolution place learning, the arena contained just four potentially rewarded locations (Figure 3B). Now, however, rather than needing to learn “*where*” the reward was hidden participants needed to learn “*how”* to find the reward (specifically, enter the arena, make a 45° rotation to the left and choose the cup against the curtained wall on the opposite side of the arena).

We found that in the LC condition where a red cup signaled the location of the reward all three groups (DS, WS, and TD children) performed similarly, and the number of individuals from each group who performed above chance did not differ. In contrast, when no red cup was present more individuals with WS (72%) and more individuals with DS (56%) performed the response learning task above chance than TD children in the same mental age range (16%; Figure 3C).

These findings are important for three main reasons. First, they show that response learning was facilitated (i.e., better than that of TD children) in individuals with WS and DS thus suggesting that impaired hippocampal function may lead to enhanced striatum-dependent behaviors in individuals with intellectual disability. Interestingly, individuals with DS showed less impairment on hippocampal-dependent tasks than individuals with WS (individuals with DS were only impaired on high-resolution but not low-resolution spatial tasks) and accordingly they also showed less facilitation on the response learning task than individuals with WS. Second, they show that not all types of spatial processing are impaired in WS and thus that not all types of spatial competencies should be considered as equivalent. Finally, they suggest that compensatory strategies can be tailored to different neurodevelopmental syndromes in order to target specific cognitive capacities that are likely to be preserved, or that can be empirically demonstrated to be preserved, in each individual.

### Path Integration and Cognitive Mapping Capacities in DS and WS

Although these findings convincingly showed that as compared to TD children in the same mental age range the majority of individuals with DS possess relatively preserved low-resolution topological allocentric coding, relatively impaired high-resolution metric allocentric coding, and somewhat facilitated response learning, we wondered whether these preserved allocentric capacities in DS were sufficient to allow these individuals to create cognitive maps. Even though theoretical and neurophysiological evidence strongly suggests that cognitive maps are subserved by the same spatial representational system that is needed to solve our open-field allocentric spatial memory task, the ability to take shortcuts to successfully navigate in the environment has come to be regarded as hallmark evidence for the existence of cognitive maps (20, 21). We also wondered whether the impaired allocentric abilities observed in the large majority of individuals with WS may be due to corrupted visual input from abnormal dorsal visual stream processing that is characteristic in this syndrome (38–40). If so, precluding corrupt visual input might allow the hippocampus to accurately process non-corrupted spatial information derived from other sensory sources and thus generate allocentric representations.

In order to answer these two questions we designed an experiment to assess the ability of blindfolded individuals with DS, individuals with WS and TD children in the same mental age range to use path integration to create and use egocentric and allocentric spatial representations to navigate in their environment in the absence of visual input (41). Our study included 19 individuals with DS (mean MA 5.57 years), 18 individuals with WS (mean MA 5.89 years) and 28 TD children (mean CA 6.91 years). The position and movement of the participants in the room were recorded using radio frequency tags attached to each shoulder (Noldus TrackLab system, Wageningen, Netherlands).

**Figure 4:**
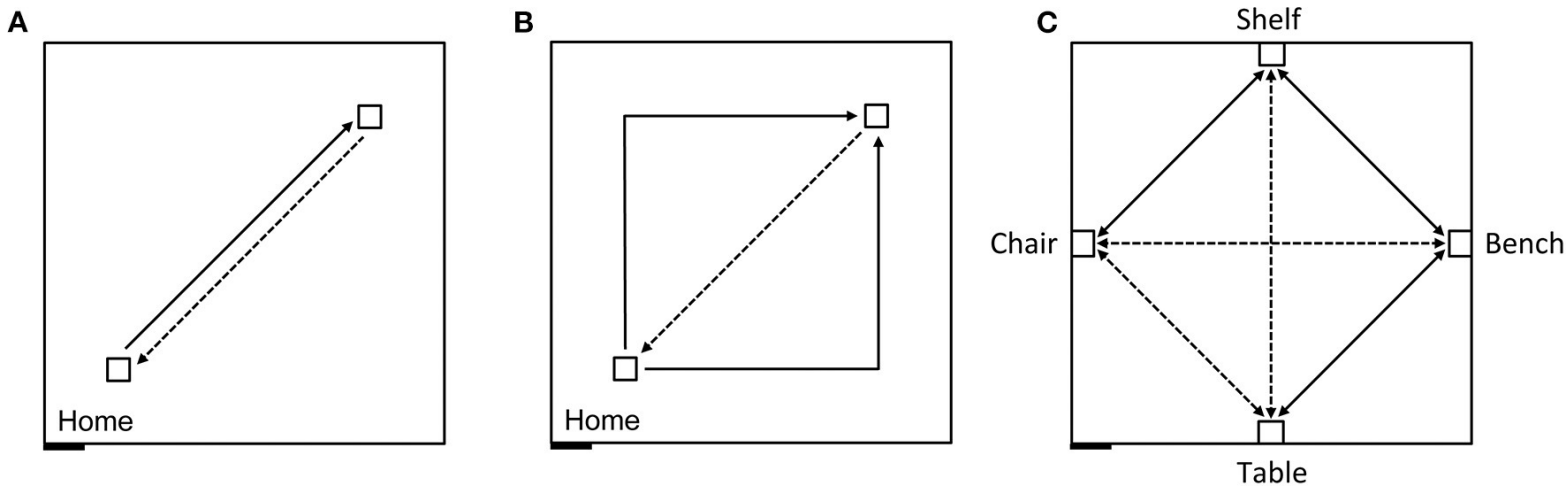
Schematic representation of the experimental design carried out in an 8 × 8 m testing room. Solid lines indicate guided trajectories; dashed lines indicate direct paths that participants were verbally requested to make. **(A)** Homing task, straight paths: 7 m straight line guided trajectory, 7 m return path. **(B)** Homing task, angled paths: 10 m angular guided trajectory with a right or left turn (5 + 5 m), and 7 m return path. **(C)** Allocentric task: Guided routes (solid) and novel routes (dashed) between four objects. The paths between the bench and the chair, and between the table and the shelf were 7 m long; the other paths were 5 m long. Figure modified from Bostelmann et al. (41).

In order to first describe the ability of participants to create and use an egocentric representation to return to a starting location (home) in the absence of vision we guided blindfolded participants on a straight outward 7 m trajectory in an 8 × 8 m room (Figure 4A) and then asked them to “go home,” which would ideally require a 180° turn and a 7 m walk. The second phase was a triangle completion task where participants were guided on an outward 10 m trajectory with a 90° left or right turn in the middle (after 5 m; Figure 4B) and then asked to “go home,” which would ideally require a 135° turn and a 7 m walk.

We found that 96% of the TD children and 84% of the participants with DS were able to create and use an egocentric representation to consistently return to the quadrant of the room that contained their starting point. In contrast, only 44% of the participants with WS were able to reliably return to the home quadrant. These results established that all the participants were willing and able to walk alone while blindfolded and that they were motivated to participate in the task. These results nonetheless also signaled that individuals with WS were less proficient at creating and using an egocentric spatial representation in the absence of visual information than either TD children or individuals with DS. Individuals with WS were also less proficient than when local visual cues (red cups) marked the rewarded location in the open-field arena in the low-resolution place learning and response learning tasks (where 67 and 78% of participants with WS performed above chance level, respectively).

We next tested the ability of the same participants to create and use a cognitive map to navigate and take shortcuts (41). In the same 8 × 8 m room, we placed four pieces of real-sized furniture, a bench, a shelf, a chair and a table, with one object placed at the center of each wall (Figure 4C). Participants, who had never seen these objects nor the room with these four objects in it, were blindfolded before entering the room. They were then guided three times and allowed to walk while still blindfolded by themselves three times, between the bench and the shelf, between the shelf and the chair, and between the bench and the table. The blindfolded participants were then immediately asked to go directly from the bench to the chair, from the chair to the table, from the table to the shelf, and then to make the reverse paths from the shelf to the table, from the table to the chair, and from the chair to the bench, thus taking three novel paths and their three reverse paths.

**Figure 5:**
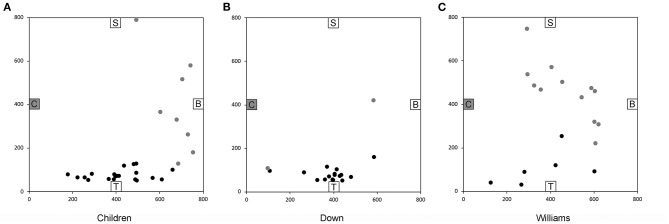
End location of participants on the novel “Chair to Table” path of the allocentric cognitive mapping task. Black circles indicate final locations in the correct quadrant. Gray circles indicate final locations in an incorrect quadrant. **(A)** 20/28 TD children ended in the quadrant of the room where the target object was located. **(B)** 17/19 individuals with DS ended in the correct quadrant. **(C)** 6/18 individuals with WS ended in the correct quadrant. Room size: 800 × 800 cm. Figure modified from Bostelmann et al. (41).

We found that across all six trials 64% of the TD children and 78% of the individuals with DS were capable of reliably navigating to the objects using novel shortcuts and thus demonstrating that they had used path integration, using only idiothetic cues, to create a cognitive map of their environment in the absence of visual information (Figure 5). In contrast, only one of 18 participants with WS (6%) was able to build a cognitive map to navigate successfully between the objects in the environment. These findings again suggested that low-resolution allocentric capacities are relatively preserved in individuals with DS, showing levels similar to those of TD children in the same mental age range, whereas these capacities are severely impaired in individuals with WS.

### Opposite Profiles of Allocentric Spatial Capacities in DS and WS

A majority of our participants were tested on both the open-field arena and the cognitive mapping task thus allowing us to make within-subject comparisons of allocentric abilities [Table 1; (41)]. By and large, the abilities of individuals with DS and individuals with WS to create and use a cognitive map without vision are consistent with our findings from the open-field arena with vision.

**Table 1:** Number of participants who passed or failed the open-field allocentric spatial memory task with one location and with vision [“Allo”; (36, 37)] and the cognitive mapping task with four objects in the absence of vision [“CogMap”; Bostelmann et al., (41)].

	**Pass Allo and Pass CogMap**	**Pass Allo and Fail CogMap**	**Fail Allo and Pass CogMap**	**Fail Allo and Fail CogMap**
TD *n* = 16	10	6	0	0
DS *n* = 18	10	4	3	1
WS *n* = 15	1	2	0	12

Of the 18 individuals with DS who participated in both tasks, 10 passed both tasks, four passed the allocentric open-field arena task but not the cognitive mapping task, and three passed the cognitive mapping task but not the open-field arena task. Thus, if we consider how many of the individuals with DS succeeded on at least one of the two allocentric spatial tasks we find that 17 of 18 participants (95%) did so, providing convincing evidence for the preservation of low-resolution allocentric spatial capacities in individuals with DS. Nonetheless, it might be somewhat surprising that three individuals with DS passed the cognitive mapping task but not the open-field task since the cognitive mapping task would likely be considered more difficult. We contend that these results suggest that cognitive mapping, even in the absence of vision, is a very natural and automatic process. When locomoting, an individual's self-motion information is continuously and automatically encoded allowing the constant updating of their position and the location of objects in the environment relative to other objects. In contrast, the allocentric open-field task requires participants to inhibit egocentric responding and/or stimulus-response impulses that encourage them to search the first visible cup encountered. Indeed, two individuals with DS who failed the open-field task adopted egocentric response strategies and either always turned right or went straight when entering the arena and then systematically searched the first cup they encountered.

Of the 15 individuals with WS who participated in both tasks, only one individual passed both tasks, whereas two individuals passed the allocentric open-field arena task but did not pass the cognitive mapping task. Thus, performance on these two tasks further confirms that allocentric spatial capacities are severely impaired, if not entirely absent, in the vast majority of individuals with WS.

### Take Home Message From Our Real-World Experiments

#### Down Syndrome

Taken together our experiments describing the allocentric and egocentric spatial memory abilities of individuals with DS in a real-world laboratory setting revealed four critical points as summarized in Table 2: (1) The large majority of individuals with DS are capable of using an egocentric representation and response learning in order to learn and remember a goal location, both in the presence or absence of visual information; (2) The large majority of individuals with DS are capable of using a low-resolution allocentric spatial representation to learn and remember a goal location, suggesting that this capacity is relatively preserved as compared to TD children in the same mental age range; (3) The majority of individuals with DS are impaired at using a high-resolution allocentric spatial representation to learn and remember three goal locations, as compared to TD children in the same mental age range; (4) Since there is significant individual variability in the spatial abilities exhibited by individuals with DS it is critical to report individual performance rather than group averages in order to accurately assess and convey the range of capacities that exist across individuals with DS.

**Table 2:** Performance of TD children and participants with Down syndrome (DS) or Williams syndrome (WS) in our real-world laboratory experiments.

	**Egocentric response learning**	**Egocentric path integration**	**Allocentric (low-resolution)**	**Allocentric (high resolution)**
TD children	Poor	Good	Good	Good
Participants DS	Facilitated	Good	Good	Impaired
Participants WS	Facilitated	Impaired	Severely impaired	Not tested

#### Williams Syndrome

Taken together our experiments describing the allocentric and egocentric spatial memory abilities of individuals with WS in a real-world laboratory setting revealed four critical points (Table 2): (1) The large majority of individuals with WS are capable of using egocentric response learning in the presence of vision to learn and remember a goal location and this ability appears to be facilitated as compared to TD children; (2) In the absence of vision fewer than half of the individuals with WS we tested were able to successfully solve an egocentric spatial task, performing significantly worse than TD children and individuals with DS in the same mental age range, thus suggesting that most individuals with WS require visual cues or beacons to successfully solve spatial tasks; (3) The large majority of individuals with WS are incapable of using a low-resolution allocentric spatial representation to learn and remember a goal location as compared to TD children in the same mental age range and individuals with DS; (4) Although we did not observe significant individual variation in the spatial abilities of the individuals with WS we tested there were nonetheless a couple of individuals who exhibited spatial abilities similar to those of TD children, again emphasizing that it is critical to report individual performance rather than group averages in order to accurately assess and convey the range of abilities that exist across individuals with WS.

## Comparison With Previous Studies With Individuals With DS

### Egocentric Tasks in Real-World Settings

To our knowledge, aside from the unpublished study by Mangan (27) and our own studies (31, 37, 41) only one other study has investigated large-scale spatial memory abilities of individuals with DS in a real-world setting. Meneghetti et al. (42) asked individuals with DS and MA-matched TD children to reproduce an egocentric 1–7-step route consisting of sequential moves forward, right or left on a 4 × 4 floor matrix comprising 16 squares of 50 × 50 cm separated by 10 cm gaps. Participants learned the route to be reproduced by either studying a map of the route or observing an experimenter take the same route.

In agreement with our findings, they found that individuals with DS performed as well as MA-matched TD children. Both individuals with DS and TD children performed better in the condition where they could observe the experimenter walking the route than in the condition where they studied the route on a map suggesting that individuals in this mental age range are not as competent in spatial tasks demanding a transfer from a schematic representation to the real world as they are in reproducing a route that they observed someone else take. In sum, studies investigating the egocentric spatial abilities of individuals with DS in the presence and absence of visual information are coherent and suggest that egocentric spatial capacities in the real world are relatively preserved in DS and similar to those of TD children in the same mental age range.

### Egocentric Tasks in Virtual Reality

A number of studies have investigated the abilities of individuals with DS to use egocentric representations to navigate in virtual environments and our results are for the most part consistent with their findings. Whereas, studies in virtual reality have found that the majority of individuals with DS are capable of learning routes using egocentric strategies, the performance of participants with DS is not always comparable to that of MA-matched TD children and the specific learning strategies used by the different groups of participants may differ (43–47).

In two related studies, Courbois et al. reported that individuals with DS can learn two different routes during a learning phase comprising two guided forward and backward trials, and up to 10 unguided trials with direct feedback provided by removable barriers that would appear to prevent participants from going further down a path after an incorrect choice (44, 46). Although each route required four changes of direction (participants used the arrows on a computer keyboard to navigate), the two forward routes could be learned and distinguished by remembering a sequence of only two different turns (left-right vs. right-left), since the initial segment of both routes was the same from the starting point, and the last segment required the same change in direction (turn right) to find the goal. This was also the case for the return routes since the initial and terminal segments required the same directional changes for both routes. Alternatively, participants could also learn to rely on the landmarks located along each path to reach the destination. For example, they could adopt a simple beacon-following strategy by approaching individual landmarks one at a time without necessarily remembering their order, or they could learn the associations between viewpoint-dependent landmark views and directional changes for the forward and backward paths. Nevertheless, these strategies were congruent so participants could rely on one or the other, or a combination to learn the routes. When asked to discriminate between landmarks placed along the paths from landmarks placed elsewhere in the virtual maze, without having to remember their specific location, participants with DS correctly remembered fewer landmarks (average: 10.5) than TD participants matched for MA (average: 13.0) or CA (average 13.5) (44).

Using a similar experimental design, Davis et al. (45) found that participants with DS needed more trials to learn the routes, committed more errors and remembered fewer landmarks located at choice points than TD children, although they remembered the same number of landmarks that were not located at choice points. Finally, using a virtual environment replica of a neighborhood of the city of Bordeaux, N'Kaoua et al. (47) found that individuals with DS had lower scores than MA-matched TD children in wayfinding performance, free recall of landmarks encountered during navigation through the neighborhood (although there were no group differences in landmark recognition), and a landmark ordering test.

Altogether these findings suggest that as compared to TD children, individuals with DS are more likely to rely on a sequence of directional changes to learn routes and may pay less attention to environmental landmarks or have more difficulty in associating those landmarks with specific spatial locations or behavioral responses. TD children's ability to rely both on the sequence of directional changes and their consistency with environmental landmarks at choice points or along the path may underlie their more efficient route learning (48). It is thus possible that the absence of visual guidance cues in our allocentric spatial studies, cues that would normally facilitate the performance of TD children as compared to individuals with DS, enabled participants with DS to perform similarly to MA-matched TD children (37, 41).

### Allocentric Tasks in Real-World Settings

To our knowledge, other than the unpublished study by Mangan (27) no other study investigated the real-world allocentric spatial abilities of individuals with DS. Our findings in two low-resolution allocentric tasks, the allocentric open-field task and the cognitive mapping task, were consistent in showing that 75–95% of individuals with DS are capable of creating and using an allocentric spatial representation to orient and navigate in their environment (37, 41). In order to succeed on these tasks participants must be able to create an allocentric representation (a.k.a., a cognitive map) of their surrounding environment that incorporates idiothetic cues, in the presence of vision in the open-field arena task, and in the absence of vision in the cognitive mapping task. Since both tasks require the use of an allocentric spatial representation it is logical to presume that they are subserved by at least some of the same neural substrates.

We were able to test 16 TD children on both allocentric tasks and found that whereas all 16 passed the open-field allocentric task, only 10 passed the cognitive mapping. Importantly, however, the performance of the TD children on the cognitive mapping task was not correlated with age thus supporting previous findings that allocentric representations can be created by TD children from around 2 years of age (28–30, 49, 50). It thus stands to reason that the variability in performance in TD children in this age range has to do with factors other than allocentric spatial competence. Of the 18 participants with DS who were tested on both tasks, 14 passed the open-field task, whereas 13 passed the cognitive mapping task. Surprisingly, of the 13 participants with DS who passed the cognitive mapping task, three failed the open-field task with one location, a finding which, similar to TD children, may highlight the influence of factors other than allocentric spatial competence such as poor comprehension of the goals of the task or impaired inhibitory processes.

In contrast to their relatively preserved performance on low-resolution allocentric spatial tasks we found that individuals with DS were impaired, as compared to TD children, on a high-resolution allocentric spatial task in the open-field arena (31). This dissociation in the performance of individuals with DS on the low-resolution and high-resolution tasks suggests that distinct cognitive processes and/or neural substrates are implicated in solving these two tasks. We will discuss evidence supporting this hypothesis in the last section of this manuscript.

In sum, both theoretical and empirical evidence support the view that successful performance in the allocentric open-field arena and the cognitive mapping tasks depends on the ability to form and use a low-resolution allocentric spatial representation of the environment. As compared to MA-matched TD children, our data show that this ability is preserved in the vast majority of individuals with DS.

### Allocentric Tasks in Virtual Reality

Although a number of studies reported an impaired ability of individuals with DS to demonstrate allocentric spatial competencies or configural knowledge of landmark locations using virtual reality paradigms, no study has provided unequivocal evidence of a specific impairment as compared to MA-matched TD children (43, 44, 46, 47). The main reason for this lack of conclusive evidence is the fact that none of these studies actually demonstrated that TD children themselves relied on allocentric or configural spatial representations to solve these tasks. In all the virtual reality studies discussed above in the context of wayfinding or route learning, alternative strategies were likely used by TD children during the tests designed to reveal allocentric or configural learning. For example, when participants were asked to take a shortcut between two locations, TD children required several trials before following the most direct route (44, 46). This suggests that TD children had not acquired sufficient configural or allocentric knowledge concerning the environment during initial learning to enable them to take the shortest route on the very first shortcut trial. Instead, the children's behavior is more consistent with them learning a new route during these free-exploration trials.

Similarly, Pennington et al. (51) tested individuals with DS and MA-matched TD children on a virtual Morris water maze task in which participants used a joystick to navigate in the environment and could use distal visual cues to remember the location of an invisible platform. Evidence of allocentric spatial mapping was assessed during a probe trial in which the platform was removed and the participants were expected to continue searching for the target throughout the duration of a 90-s trial. Individuals with DS spent on average 17 ± 9% of the time searching for the platform in the quadrant of the maze where the platform was located whereas MA-matched TD children spent 30 ± 21% of the time in the quadrant of the platform (chance performance is at 25%). It is important to keep in mind, however, that other factors such as motivation, confidence or the drive to explore other parts of the environment to look for the platform when it was not located where the participant expected it to be may also influence the time spent searching in the target quadrant. Moreover, the lack of individual data makes it impossible to determine the proportion of individuals with DS and TD children who searched for the missing platform where it was located previously if even for a short period of time, and thus demonstrated evidence of allocentric spatial learning. In a subsequent study using the same paradigm, Edgin et al. (52) failed to reveal any difference in the time spent searching for the platform in the target quadrant between individuals with DS (26.73 ± 19.83%) and MA-matched TD children (20.69 ± 21.19%) thus raising doubts about a global and reliable impairment in allocentric spatial learning and memory in individuals with DS in this virtual task.

Finally, Toffalini et al. (53) evaluated the ability of individuals with DS and MA-matched TD children to learn the locations of five local landmarks distributed at the four corners and along one of the walls of a square arena. Participants discovered four different virtual environments by either viewing a video from the perspective of a person walking through the environment, described as route learning, or observing footsteps following the same path from an aerial perspective, described as survey learning. Although some subtle differences were reported in the performance of the two groups in the different learning conditions, statistical analyses suggested that individuals with DS performed as well as TD children when asked to place the landmarks at their approximate locations on a layout of the environment. However, experimental data were not described in sufficient detail to provide evidence of configural knowledge, or to evaluate the performance of individual subjects.

In sum, none of the previous studies carried out in virtual reality provided reliable evidence regarding the ability or inability of individuals with DS to build an allocentric spatial representation of their environment. In contrast, our studies have provided unequivocal evidence that a large majority of individuals with DS (75–95%) are able to create and use a low-resolution allocentric representation to successfully orient and navigate in real-world environments (37, 41).

## Comparison With Previous Studies With Individuals With WS

### Egocentric Tasks in Real-World Settings

Two previous studies evaluated the ability of individuals with WS to perform efficient spatial searches in real-world paradigms. In a study by Smith et al. (54) which specifically evaluated egocentric search strategies, participants searched for a target light amongst an array of between 5 and 20 decoy lights arranged in an irregular pattern on the floor in a square arena completely surrounded by black curtains. Two participants with full WS deletions in the critical region took longer to find the target light and made more errors by revisiting locations that had been previously visited on the same trial than TD control participants and two WS individuals with partial deletions, thus showing that their search was disorganized and that they failed to egocentrically encode previously visited locations.

In a study by Foti et al. (55) participants could use either egocentric or allocentric strategies to efficiently search for nine rewards hidden under nine inverted buckets arranged in either a cross formation, a 3 × 3 square or 3 triangular-shaped clusters of 3. Participants with WS performed less well than MA-matched TD children on all measures. They made more total errors (re-visits or no-visits), and had fewer errorless trials and lower spatial spans, thus also demonstrating disorganized searching and an inability to encode previously visited locations in either an egocentric or allocentric representation. In sum, both of these studies showed that individuals with WS are impaired at performing efficient egocentric search strategies in tasks that require strict sequential searches of multiple visually identical (e.g., non-distinct) and closely apposed spatial locations which require high-resolution spatial discrimination abilities.

In contrast, we showed that individuals with WS were far better than TD children and better than individuals with DS at using an egocentric response strategy to find one goal in a low-resolution open-field arena. It is possible that in our paradigm individuals with WS were able to combine a response strategy with a visual guidance strategy to succeed. Thus, when individuals with WS entered the arena they performed the same fixed motor response: they turned slightly to the left and looked for the cup next to the curtain on the opposite side of the arena and walked toward that cup. In this task, although the cup was not visually distinct since there were three other identical cups in the arena, it was spatially distinct since it was not near any other cup and could thus be targeted visually when combined with a fixed motor response that would orient the participant in the direction of the rewarded cup on every single trial.

Although participants were afforded numerous trials, 10 pairs of trials with the local cue (red cup) present and 10 pairs of trials with no local cue present in order to learn and repeat the fixed motor response, many participants with WS learned this motor response on the first two trials (since the red cup was present) and made very few errors afterwards, as if this type of navigation was their default strategy. This hypothesis is supported by findings from a study by Farran et al. (56) showing that even though individuals with WS performed less well than MA-matched TD children they were nonetheless able to learn a new 1 km long route including 20 choice points (left, right, straight ahead) through an unfamiliar environment. When the participants with WS were given repeated experience walking the route and verbal instructions including directional information and information about key landmarks along the routes their performance improved. These findings suggest that during everyday orientation and navigation, individuals with WS may rely on fixed behavioral responses combined with visual guidance cues, and that during autonomy training these individuals should be taught to rely on learned sequential egocentric responses combined with viewpoint-matching of sequentially visible landmarks along the route.

In contrast, in our egocentric path integration task where participants were blindfolded and led on straight or angled paths and asked to return to their starting point, individuals with WS performed worse than TD children and individuals with DS. Since in this task there were no visual cues participants could only rely on idiothetic cues. These findings show that individuals with WS have difficulty in integrating idiothetic cues and further suggest that visual cues may be the primary information used by individuals with WS when they successfully orient and update their spatial location in the world.

In sum, individuals with WS can likely use egocentric response learning in combination with memorized sequences of landmarks to navigate routes in the real world. It must be recognized, however, that this type of navigation is not flexible and is vulnerable to perturbations in the environment such as when landmarks are removed or obscured or when detours modify the route.

### Egocentric Tasks in Virtual Reality

Studies carried out in virtual reality also show that a majority of individuals with WS are capable of route learning using landmarks located along the path (46, 57, 58). In a study using a design similar to the one described above for individuals with DS (44), Farran et al. (46) reported that about two thirds of individuals with WS can learn at least one of two different routes requiring four changes in direction. On average individuals with WS performed similarly to individuals with DS and both groups made about three times more errors while learning the routes than MA-matched TD children.

Using a differently shaped virtual environment, Broadbent et al. (58) showed that individuals with WS demonstrated a reliance on visual landmarks for route-learning and failed to learn a route containing six changes in direction that did not contain landmarks and thus depended uniquely on the ability to build a memory representation of the sequence of left-right turns. These findings are consistent with those reported by Broadbent et al. (57) for the learning of a route comprising four directional changes in a cross-maze virtual environment. Following a guided trial during which a grass path indicated the route to follow, participants were given up to 10 free-exploration trials to learn the route. Participants were then placed at a different starting location and asked to navigate to the correct exit. During these spontaneous strategy tests slightly more than 50% of TD 5–10-year-old children employed an egocentric strategy consisting of the correct succession of left and right turns corresponding to the learning trials. In contrast, about 40% of individuals with WS relied on a so-called “mixed” strategy which upon close inspection of the experimental design may potentially reflect some learning of the individual landmarks along the path.

Finally, in another study Farran et al. (59) showed that as compared to MA-matched TD children, individuals with WS spent as much time looking at landmarks situated along a path but less time looking at distal landmarks. Thus, in contrast to what has been shown for individuals with DS who do not benefit from the presence of environmental landmarks but can learn a sequence of directional changes, individuals with WS appear to rely more heavily on local environmental landmarks. This may include remembering the sequence of landmarks and associated changes in direction or using a beacon navigation strategy from one landmark to the next without remembering directional changes at choice points.

### Allocentric Tasks in Real-World Settings

We investigated allocentric spatial abilities in individuals with WS using two very different tasks. The low-resolution open-field arena in which participants had access to coherent visual and idiothetic information in order to learn and remember the location of one reward, and the cognitive mapping task in which blindfolded participants had access to only idiothetic information in order to learn the locations of four different pieces of furniture in a large room. We found that individuals with WS were severely impaired on both of these tasks as compared to MA-matched TD children and individuals with DS (36, 41). As discussed above, this empirical evidence is consistent with theoretical arguments that performance on both tasks depends on the ability to form and use a low-resolution allocentric spatial representation of the environment. Our findings thus provide unequivocal evidence arguing for the severe impairment in, or absence of, the ability of the vast majority of individuals with WS to create or use an allocentric spatial representation of their environment.

Our findings support those of the experiment by Farran et al. (56) in which individuals with WS were able to learn to reproduce a walking route through a natural environment. However, whereas MA-matched TD children were also able to learn the spatial relationships between environmental landmarks as shown by their ability to point accurately in the direction of several unseen landmarks from different points along the route, a competence that requires an allocentric representation of the surrounding environment, individuals with WS were not able to do so. We note that a number of other studies employing real-world paradigms have also suggested deficits in allocentric spatial processing in individuals with WS but because success in these paradigms also depended on other cognitive processes such as the ability to understand complex verbal instructions, mental rotation and working memory (55, 60, 61) they do not provide unequivocal evidence that allocentric spatial learning *per se* is impacted in WS. In contrast, our findings from two different paradigms provide unequivocal evidence that allocentric spatial processes are severely impaired, if not abolished, in a large majority of individuals with WS (36, 41).

### Allocentric Tasks in Virtual Reality

Several studies have reported an impaired ability of individuals with WS to demonstrate allocentric or configural knowledge of landmark locations in virtual reality paradigms (46, 57, 58). Nevertheless, as discussed above these studies cannot be considered as providing unequivocal evidence of a specific allocentric impairment as compared to MA-matched TD children since TD children tested in these paradigms did not conclusively demonstrate that they actually relied upon an allocentric or configural spatial representation to solve these tasks.

For example, Broadbent et al. (57) showed that only between 20 and 30% of TD children between 5 and 8 years of age, which corresponds to the mental age of individuals with WS, may have used an allocentric strategy to solve a cross-maze task, and <50% of 10-year-old TD children may have done so. Such poor performance by TD children makes comparisons of the performance of individuals with WS relatively uninformative and raises the question as to whether wayfinding tasks designed to test allocentric abilities in virtual environments are even valid in children and individuals with intellectual disabilities with mental ages under 10 years. Wayfinding in virtual environments demands the combined use of numerous cognitive capacities. For example, the participant must be able to understand that they are in an environment that is a proxy for the real world which retains certain real-world physical properties that can be used to create a cognitive map of the environment. It does not seem to be the case that the majority of children under 10 years of age either can or readily do this. In contrast, in the real world children from 2 years of age are capable of using an allocentric representation, and from at least 5 years of age are capable of building a cognitive map in absence of visual information (20, 29, 30).

In sum, we cannot stress enough that whereas experiments in virtual environments that succeed in demonstrating egocentric or allocentric spatial competencies in certain populations may be conclusive, a failure of participants to succeed in virtual environments cannot be considered as evidence of the absence of these spatial capacities in the real world. This is especially true for individuals with neurodevelopmental disorders, or in paradigms where the control group of MA-matched TD children fails to demonstrate the target ability. In contrast, we have shown that in real-world laboratory settings nearly all TD children from 3 years of age and ~95% of individuals with DS are capable of creating and successfully using allocentric spatial representations of their environment (29, 30, 37, 41), numbers that differ substantially from those observed in virtual reality settings. It is thus critical that researchers who intend to use virtual reality paradigms to investigate spatial competence in children and individuals with intellectual disability carefully evaluate the ecological validity of their paradigms and their results.

## Neurobiology of Spatial Learning and Memory in DS and WS

When carefully evaluated, our findings and the findings from previous real-world and virtual reality experiments provide a consistent perspective. We now endeavor to explain how the differential impairments observed in the various experiments summarized above can be used to infer the neurobiological substrates that may be relatively impacted or preserved in individuals with DS or WS, as compared with TD children within the same mental age range, and thus may underlie the spatial cognitive profiles observed in these two neurodevelopmental syndromes.

### Egocentric Representations

We begin by examining the neuroanatomical substrates that subserve egocentric homing, which was necessary for our blindfolded participants to return to their starting point after being led on straight or angled outward journeys. Homing requires the use of path integration to keep track of the changes in direction and the distance traveled during the outbound journey in order to return to a starting point using the most direct route (62, 63). Path integration relies on information generated from self-motion, including vestibular and proprioceptive information and efferent motor copies, and can be used to build both egocentric and allocentric representations. Egocentric representations, however, need not contain contextual spatial information capable of placing an individual in a particular location in relation to other environmental landmarks (64).

The directional signal of path integration is provided by so-called head-direction cells found in several brain structures including the dorsal tegmental nucleus, the mammillary nucleus, the thalamus, the presubiculum, the parasubiculum, and the retrosplenial and entorhinal cortices (21, 65, 66). In our egocentric homing task, individuals with DS exhibited relatively preserved performance (i.e., similar to MA-matched TD children), suggesting that the brain structures or circuits subserving path integration exhibit relatively preserved function in this syndrome. In contrast, homing performance was worse in individuals with WS, suggesting that the brain structures or circuits subserving path integration are more selectively impaired in WS.

### Response Learning

Response learning requires participants to learn fixed stimulus-response representations of behavioral performance, also known as habits (21). Thus, when participants entered the arena during our response learning task, the specific viewpoint-dependent representation of the arena with a visible potential goal location likely triggered the associated motor response: “*Walk four meters slightly to my left to reach the goal located at the mid-point along the opposite wall*.”

Response learning is dependent on the dorsal striatum (9), as well as visual areas necessary to process individual objects or information including the perirhinal and rostral entorhinal cortices (67–69), visual areas necessary to process viewpoint-dependent scenes including the parahippocampal, retrosplenial and caudal entorhinal cortices, and medial parietal regions (21). Response learning has been shown to be facilitated in the presence of hippocampal dysfunction (9). In our response learning task, individuals with DS and individuals with WS exhibited better performance than MA-matched TD children, suggesting that the neurobiological substrates subserving response learning are relatively intact in these two syndromes and that impaired hippocampal function contributes to improved performance in striatal-dependent tasks in these individuals (36, 37, 70).

### Route Learning

Route learning tasks with landmarks may be solved by simply relying on a beacon-guidance strategy. Participants can simply approach familiar landmarks without necessarily remembering the chronological sequence of these landmarks along the route, since the order in which they are encountered is usually determined by the testing environment. Alternatively, participants may learn to associate specific landmarks with directional changes, also without having to remember the sequence in which the landmarks are encountered. As described above for our response learning task, route learning also appears to depend primarily on the caudate nucleus of the dorsal striatum (10, 11, 21, 71), as well as cortical areas necessary to recognize viewpoint-dependent landmarks and visual scenes including the parahippocampal and retrosplenial cortices, and medial parietal regions (21, 72).

The experiments described above have shown that individuals with DS can generally learn routes in both real and virtual environments, but they do not appear to benefit from the presence of environmental landmarks. Impaired landmark use in individuals with DS may be linked to dysfunctions in cortical areas contributing to the processing of local features related to individual objects or landmarks, including the perirhinal and rostral entorhinal cortices (67–69). These areas may further contribute to the integration of individual objects into a local representation of the environment (73, 74), and thus impact the precision and capacity of spatial memory (75). However, impaired landmark use in individuals with DS may also be linked to impaired working memory capacity, which would limit their ability to remember as many landmarks or landmark-response associations (e.g., turn left at the church, turn right at city hall) as TD children. Indeed, we have previously shown that memory capacity is linked to memory precision (75). Memory precision, in turn, has been shown to depend on pattern separation in the dentate gyrus of the hippocampus (76–78), which appears to be more highly impacted in DS than other regions of the hippocampus (see below).

Route learning tasks without landmarks are typically realized in virtual or real-world labyrinth-like environments. In these tasks, participants must learn a sequence of changes of directions (left-right), without necessarily needing to define the magnitude of each of these changes in orientation (angles), nor the absolute distance traveled between choice points, since these parameters are constrained by the environment. Whereas, this ability could be dependent on striatal-dependent motor learning, as well as the ability to encode the directional changes verbally, it does not require that landmark or visual-scene information be integrated with directional information derived from path integration. This type of route learning may thus depend primarily on the striatal-learning system (10, 11, 21, 71), although other brain regions including the insula/ventrolateral prefrontal cortex, right anterior prefrontal cortex, and cerebellum may also play a role (10, 79).

Individuals with DS consistently exhibit relatively preserved performance on sequential learning and implicit learning tasks (4, 80), which are also thought to be subserved by the striatal-learning system (81, 82). Similarly, individuals with DS exhibit relatively preserved performance on route learning tasks without landmarks, again consistent with their preserved homing and response learning capacities.

Surprisingly, even though individuals with WS exhibited facilitated response learning in our experiments, they consistently exhibit more impaired performance on route learning tasks in absence of landmarks. Altogether, for individuals with WS, the combination of impaired homing and route learning in the absence of landmarks, with relatively preserved route learning in the presence of landmarks and response learning, suggests that they may use a beacon-guidance strategy as their primary means of navigation in both virtual and real-world environments, even when a response strategy may result in better performance.

### Allocentric Representations

Conceptually, allocentric spatial representations and cognitive maps are considered to be synonymous (19, 21, 83), and thus dependent on the same brain regions. In this manuscript we have used the two different terms only to clearly distinguish between the two experimental paradigms and the behavioral evidence that was provided by these experiments. In the allocentric open-field spatial task with vision (36), participants must determine the location of the goal in relation to distant objects present in the environment (14, 18, 19, 72, 83). Even though the function of the hippocampus is not limited to allocentric spatial processing (19), numerous studies carried out in rats, monkeys and humans have shown that the hippocampus plays a fundamental role in this ability (17–19, 22, 72).

As described above, path integration can be used to construct an allocentric representation of the environment which contains contextual spatial information capable of placing an individual in a particular location in relation to other environmental landmarks (20, 84, 85). Accordingly, the brain structures contributing to homing, such as the dorsal tegmental nucleus, the mammillary nucleus, the thalamus, the presubiculum, and the parasubiculum (21, 65, 66) will also be involved in the elaboration of allocentric spatial representations of the environment (83). In addition, proper functioning of cortical areas including the parietal, retrosplenial, parahippocampal, and entorhinal cortices will contribute to the integration of visual and idiothetic information into a multimodal spatial representation (13, 14, 18, 19, 21, 22, 35, 68, 73, 83, 86, 87). Accumulating evidence also suggests that the cerebellum may be involved in the integration of multimodal self-motion information (88) as well as other aspects of spatial learning (24).

In the cognitive mapping task without vision, participants must use path integration exclusively to learn the spatial relations between four different objects that cannot be perceived simultaneously, in order to build an allocentric spatial representation of the environment enabling them to navigate flexibility and take never-experienced paths or shortcuts between these objects, the hallmark evidence for cognitive maps (20, 21, 89, 90). Accordingly, the same brain structures contribute to the elaboration of cognitive maps as was described for allocentric representations, except that visual information is not available in our cognitive mapping task.

However, it is also important to consider that although allocentric spatial representations depend on the hippocampal formation, different functional capacities are thought to arise from complementary but dissociable spatial computations within different hippocampal regions (91). Basic low-resolution allocentric spatial processing depends on a direct projection from the entorhinal cortex to CA1 (33, 35). In contrast, imaging studies in humans, neurophysiological studies in rats, and computational models, have established that pattern separation, the ability to discriminate similar items or closely apposed locations is subserved by the dentate gyrus and CA3 (76–78). Accordingly, disrupting the CA3 input to CA1 results in decreased spatial tuning of CA1 place cell activity but does not abolish place cell activity itself (33, 34). This suggests that the dentate gyrus-CA3 functional circuit is necessary for building high-resolution spatial representations but not low-resolution spatial representations.

In addition, experiments in rats have shown that the lateral entorhinal cortex [which corresponds to the rostral entorhinal cortex in primates, including humans (92)] contributes to the integration of individual objects into a local spatial representation (93). Since this part of the entorhinal cortex receives major projections from the perirhinal cortex, which is involved in the processing of object-related information, it has been previously suggested that these areas also contribute to the elaboration of high-resolution spatial representations of the environment (73, 74). In turn, the precision of the memory for individual locations might determine the capacity of hippocampus-dependent memory for spatial and non-spatial information (19, 75).

Individuals with DS exhibit relatively preserved low-resolution allocentric spatial and cognitive mapping abilities [respectively, 78 and 74% of tested individuals; (37, 41)]. In contrast, a majority of individuals with DS (85%) were unable to build and use a high-resolution allocentric spatial representation in the presence of visual information (31). Neuroimaging findings in individuals with DS have reported structural and functional abnormalities in a number of different brain regions (94). However, some neuroimaging and neuroanatomical evidence is particularly relevant in light of our current behavioral findings. Although overall hippocampal volume has been shown to be reduced in adults (95, 96) and children (97) with DS, neuropathological findings suggest that the dentate gyrus may be relatively more impacted (98). Thus, behavioral data showing greater deficits on tasks requiring higher levels of spatial resolution, and perhaps poorer memory for and integration of visual landmarks to support higher-resolution spatial representations, in the presence of facilitated response learning, are consistent with these neuropathological findings.

Individuals with WS exhibit severely impaired low-resolution allocentric spatial and cognitive mapping abilities [respectively, only 17 and 6% of tested individuals exhibited these abilities (36, 41)]. Neuroimaging in individuals with WS have reported structural and functional abnormalities in many different brain regions (99–102), but in particular in the dorsal visual stream including the parietal and lateral occipital cortices (39, 40, 103), and in the hippocampal formation (103, 104). Interestingly, the parietal cortex is reciprocally connected with the cingulate and retrosplenial cortices, which are themselves reciprocally connected with several regions of the medial temporal lobe including the entorhinal and parahippocampal cortices, the presubiculum and the parasubiculum (67, 69, 105–107).

Our behavioral findings of impaired homing and allocentric spatial/cognitive mapping abilities in conjunction with facilitated response learning in individuals with WS are consistent with specific dysfunctions in both egocentric processing associated with the dorsal visual stream, as well as allocentric processing associated with the hippocampal formation and directly interconnected brain regions, in particular the retrosplenial cortex. Importantly, the present findings rule out the possibility that spatial impairments in WS arise from the active integration of a corrupted signal from the dorsal visual stream into higher-order regions fundamental for spatial processing that function even in the absence of vision in typically developed individuals. Rather, these findings raise the possibility that integration of corrupted dorsal visual stream information during development permanently and fundamentally changes how spatial information is processed in the hippocampus throughout life in WS.

## Conclusion

Our studies using real-world laboratory paradigms in which participants locomote freely provide a new and comprehensive perspective on the specific profiles of spatial learning and memory abilities of individuals with DS and WS. As compared to TD children in the same mental age range, the majority of individuals with DS exhibited preserved low-resolution egocentric and allocentric spatial learning and memory abilities, but impaired high-resolution spatial abilities. Interestingly, this spatial memory profile is consistent with neuroanatomical evidence suggesting that hippocampal dysfunction may be linked to abnormalities in the dentate gyrus-CA3 region in DS. In contrast, nearly all individuals with WS exhibited severely impaired low-resolution allocentric spatial learning but facilitated egocentric response learning. This spatial memory profile is consistent with neuroimaging data suggesting impaired processing in the dorsal visual stream and hippocampal dysfunction in WS. Together with work from other laboratories, our findings suggest that in order to navigate in their environment most individuals with DS may use either egocentric route learning that does not integrate many individual landmarks, or low-resolution allocentric spatial representations that encode the relationships between different locations (i.e., cognitive mapping). In contrast, most individuals with WS may use visually and verbally encoded landmarks as beacons to learn routes but they are unable to build or use low-resolution allocentric or configural representations of the environment.

## Author Contributions

PB and PL were responsible for the conception and design of the work, acquisition, analysis and interpretation of the data, and writing of the manuscript. All authors contributed to the article and approved the submitted version.

## Conflict of Interest

The authors declare that the research was conducted in the absence of any commercial or financial relationships that could be construed as a potential conflict of interest.
